# Multidrug resistant *Vibrio cholerae* O1 from clinical and environmental samples in Kathmandu city

**DOI:** 10.1186/s12879-015-0844-9

**Published:** 2015-02-27

**Authors:** Upendra Thapa Shrestha, Nabaraj Adhikari, Rojina Maharjan, Megha R Banjara, Komal R Rijal, Shital R Basnyat, Vishwanath P Agrawal

**Affiliations:** Research Laboratory for Biotechnology and Biochemistry (RLABB), Sanepa, Lalitpur Nepal; Department of Microbiology, Kantipur College of Medical Science (KCMS), Sitapaila, KTM Nepal; Central Department of Microbiology, Tribhuvan University, Kirtipur, KTM Nepal

**Keywords:** *Vibrio cholerae* O1 Classical, Resistant profile, Multidrug resistant, Cholera toxin *(ctx)* gene

## Abstract

**Background:**

Cholera, an infectious disease caused by *Vibrio cholerae,* is a major public health problem and is a particularly burden in developing countries including Nepal. Although the recent worldwide outbreaks of cholera have been due to *V. cholerae* El Tor, the classical biotypes are still predominant in Nepal. Serogroup O1 of the *V. cholerae* classical biotype was the primary cause of a cholera outbreak in Kathmandu in 2012. Thus, this study was designed to know serotypes and biotypes of *V. cholerae* strains causing recent outbreak with reference to drug resistant patterns. Moreover, we also report the toxigenic strains of *V. cholerae* from both environmental and clinical specimens by detecting the *ctx* gene.

**Methods:**

Twenty four *V. cholerae* (n = 22 from stool samples and n = 2 from water samples) isolated in this study were subjected to Serotyping and biotyping following the standard protocols as described previously. All of the isolates were tested for antimicrobial susceptibility patterns using the modified Kirby-Bauer disk diffusion method as recommended by CLSI guidelines. The screening of the *ctx* genes (*ctxA2-B* gene) were performed by PCR method using a pair of primers; C2F (5′-AGGTGTAAAATTCCTTGACGA-3′) and C2R (5′-TCCTCAGGGTATCCTTCATC-3′) to identify the toxigenic strains of *V. cholerae*.

**Results:**

Among twenty four *V. cholerae* isolates, 91.7% were clinical and 8.3% were from water samples. Higher rate of *V. cholerae* infection was found among adults of aged group 20–30 years. All isolates were serogroups O1 of the *V. cholerae* classical biotype and sub serotype, Ogawa. All isolates were resistant to ampicillin, nalidixic acid and cotrimoxazole. 90.9% were resistant to erythromycin however, tetracycline was found to be the most effective drug for the isolates. All isolates were multidrug resistant (MDR) and possessed a *ctx* gene of approximately 400 base pairs indicating the toxigenic strains.

**Conclusion:**

Hundred percent strains of *V. cholerae* were MDR possessing a *ctx* gene. It suggests that toxigenic strains be identified and proper antibiotic susceptibility testing be conducted. This will allow effective empirical therapy to be used to treat and control cholera.

## Background

Cholera is the second leading cause of mortality worldwide among children under 5 years, and is one of the main causes of morbidity in adults [1,2]. The causative agent of cholera, *Vibrio cholerae* is a genetically versatile bacterial species [3]. More than two hundred serogroups were identified on the basis of the somatic O antigens [4] among which O1 and O139 are two major virulent strains. Two biotypes of *V. cholerae* O1; classical and El Tor are the causative agents of the sixth and the seventh pandemics respectively [5]. Organisms of both biotypes of serogroup *V. cholerae* O1 are further subdivided into Serotypes; Inaba, Ogawa and Hikojima [6]. *V. cholerae* O1 is still frequently isolated from many outbreak regions of Asian countries [7]. Nepal is still a cholera endemic country where cholera outbreaks occur every year in the major cities including Kathmandu Valley causing significant morbidity and mortality [8-10].

The major virulence factors of cholera are mainly associated with the CTX genetic element which corresponds to CTX Φ (prophage), a lysogenic filamentous bacteriophage. The genetic element comprises of two gene clusters, the core and the RS2 regions. The core region contains *ctx* genes encoding the cholera toxin (CT) and five more genes encoding the required components for phage morphogenesis [7]. The toxin produced is transported extracellularly by type II secretion system disrupting the ion transport of intestinal epithelial cells [11]. The subsequent loss of water and electrolytes leads to severe secretory diarrhoea, a characteristic of cholera [12]. The presence of such genes confirms the toxigenic strains of *V. cholerae*.

The poor socio-economic status, inadequate sanitation and poor access to safe drinking water are the major predisposing factors of cholera outbreaks in many cities of Nepal. *V. cholerae* usually spreads by the faecal-oral route by ingesting faecally contaminated water or food, person to person transmission and direct contact with infected faeces as described in previous studies. Antimicrobial therapy is commonly recommended for shortening the duration or reducing the severity of symptoms as well as lessening bacterial excretion. However the problem of antimicrobial resistance among the agent continues to be alarming. Despite few studies on diarrhoeal diseases in Nepal, there is lack of adequate information on bacterial enteric pathogens and their antimicrobial resistance trend has been changed over a longer time period globally and in Kathmandu valley as well [9,13,14]. In addition, emergence of multidrug resistant *V. cholerae* isolates is a major problem in developing countries today [15]. Since most diarrhoeal diseases are treated empirically, it is important to know the susceptibility pattern of the prevalent pathogens. Hence, this study aimed to identify the toxigenic strains of *V. cholerae* isolates from clinical and environmental samples in Kathmandu city and to know their changing resistant profiles.

## Methods

### Informed consent and ethical approval

Written consent was obtained from the participants’ involved in the research study. The research ethics was approved by Nepal Health Research Council (NHRC), Kathmandu, Nepal.

### Isolation, identification and typing of *V. cholerae*

During cholera outbreak in Kathmandu city in 2012, a total of 450 stool samples from patients with diarrhoea and 30 drinking water samples from the cholera outbreak regions in the Kathmandu city were collected. The samples were enriched in alkaline peptone water (pH-8.4) at 37°C for 4–6 hours, followed by overnight culture on selective media; thiosulphate citrate bile sucrose agar (TCBS-HiMedia). The sucrose fermenting yellow colonies were subjected to biochemical tests [16] and Serotyping using kit (Mast Group and Denka Seiken, Japan) as per the kit’s instructions. The biotyping of the strains were assayed using the Polymyxin B (50 U) sensitivity test, Voges Proskauer reaction in methyl red Voges Proskauer (MRVP-HiMedia) broth medium and chicken RBC agglutination tests [6].

### Antibiotic susceptibility test

The antimicrobial susceptibility testing of the isolates to various antimicrobial disks (HiMedia: Ampicillin-10mcg, Nalidixic acid-30mcg, Ciprofloxacin-5mcg, Cotrimoxazole1.25/23.75mcg, Cefotaxime-30mcg, Chloramphenicol-30mcg, Tetracycline-30mcg and Erythromcycin-15mcg) was performed using the modified Kirby-Bauer disk diffusion method as recommended by Clinical and Laboratory Standards Institute guidelines [17]. *Escherichia coli* (ATCC, 25922) was used for the standardization of the Kirby-Bauer test for correct interpretation of the zone diameters.

### Molecular assay

PCR assay was selected as a molecular assay in this study. The genomic DNA of all isolates were extracted and purified from the aerobically grown culture in Luria Bertani (LB) broth and used for the specific PCR for the detection of *ctx* genes [18]. A pair of primers (Macrogen, Republic of Korea); C2F (5′-AGGTGTAAAATTCCTTGACGA-3′) and C2R (5′-TCCTCAGGGTATCCTTCATC-3′) were used for the gene amplifications as described by Patrick *et al.* [19]. The reaction mixture for the gene amplification was prepared in 25 μl consisting of 12.5 μl QIAGEN multiplex PCR master mix, 1 μl 10 μM forward primer, 1 μl 10 μM reverse primer, 9.5 μl distilled water and 1.0 μl of template DNA. The amplifications were performed as follows: an initial pre-denaturation at 94°C for 15 minutes followed by 35 cycles at 94°C for 30 seconds (denaturation), 60°C for 60 seconds (primer annealing), 72°C for 60 seconds (DNA extension) and a final elongation was performed at 72°C for 10 minutes on a thermocycler (Thermal cycler Perkin Elmer cetus P11966). The amplified products were fractionated by electrophoresis through 1.5% agarose gel with NEB 100 bp marker DNA which was visualized by staining the gel with ethidium bromide [19].

### Data analysis

Data were entered and analyzed using SPSS software for Windows (version 16). Chi square test was used as a statistical tool to correlate between different age groups and *V. cholerae* infection rate.

## Results

Altogether twenty four *V. cholerae* were isolated of which 91.7% (n = 22) were from patients with diarrhoea and 8.3% (n = 2) were from drinking water samples. Among the clinical isolates, 50% were isolated from adult patients of aged 20–30 years. There was significant difference in *V. cholerae* infection rate among 20–30 years aged patients as compared to other age groups (p = 0.018).

### Serotyping and biotyping of *V. cholerae*

All strains were found to be serogroup O1, serotype Ogawa and the Classical biotypes (Table 1).

**Table 1:** **Serotyping and Biotyping of** ***V. cholerae***

**Typing methods**	**Tests performed**	**Serotypes**	**No of positive strains (%)**	**Types**
Serotyping	Agglutination (Mast Group and Denka Seiken Kit**,** Japan)	Ogawa	24 (100)	Serotypes Ogawa
		Inaba	0	
		Hikojima	0	
Biotyping	Voges Proskauer		0	Classical biotypes
	Polymyxin B sensitivity		24 Sensitive (100)	
	Chicken cell agglutination		0	

### Antibiotic resistance patterns

All clinical *V. cholerae* strains were susceptible to tetracycline. However, 90.9% was susceptibility to both ciprofloxacin and chloramphenicol. The sensitivity to cefotaxime was 81.8%. All isolates were found to be resistance to ampicillin, nalidixic acid and cotrimoxazole and 90.9% isolates were resistance to erythromycin.

Among the environmental *V. cholerae* isolates, all were resistance to ampicillin, nalidixic acid, cotrimoxazole and erythromycin. Fifty percent (n = 1) isolates were resistance to chloramphenicol as well (Table 2).

**Table 2:** **General antibiotic susceptibility pattern of** ***V. cholerae*** **(total no = 24)**

**Antibiotics used (HiMedia)**	**Clinical isolates (n = 22)**		**Environmental isolates (n = 2)**	
	**No. of Resistant (%)**	**No. of Sensitive (%)**	**No. of Resistant (%)**	**No. of Sensitive (%)**
Ampicillin	22 (100)	0	2 (100)	0
Nalidixic acid	22 (100)	0	2 (100)	0
Ciprofloxacin	2 (9.1)	20 (90.9)	0	2 (100)
Cotrimoxazole	22 (100)	0 (81.8)	2 (100)	0
Cefotaxime	4 (18.2)	18	0	2 (100)
Chloramphenicol	2 (9.1)	20 (90.9)	1 (50)	1 (50)
Tetracycline	0	22 (100)	0	2 (100)
Erythromcycin	20 (90.9)	2 (9.1)	2 (100)	0

### Antibiotic resistance profiles

Five different types of resistant profiles were observed among the clinical isolates which were named as the clinical resistant type 1 to type 5 profiles. Two isolates were resistance to only three antibiotics; ampicillin, nalidixic acid, cotrimoxazole named as clinical resistant type 1 (CR_1_) profile. Higher no. of isolates; 68.2% (n = 15) were of the CR_2_ type. Similarly, the resistant types, CR_3_, CR_4_ and CR_5_ were seen in 13.6%, 4.5% and 4.5% isolates respectively (Table 3).

**Table 3:** **Antibiotic resistant profile of** ***V. cholerae*** **isolates**

**Resistant types**	**Resistant profiles**	**No. of isolates (%)**
**Clinical isolates**		
CR_1_	Ampicillin, Nalidixic acid, Cotrimoxazole	2 (9.1)
CR_2_	Ampicillin, Nalidixic acid, Cotrimoxazole, Erythromycin	15 (68.2)
CR_3_	Ampicillin, Nalidixic acid, Cotrimoxazole, Erythromycin, Cefotaxime	3 (13.6)
CR_4_	Ampicillin, Nalidixic acid, Cotrimoxazole, Erythromycin, Chloramphenicol, Ciprofloxacin	1 (4.5)
CR_5_	Ampicillin, Nalidixic acid, Cotrimoxazole, Erythromycin, Chloramphenicol, Ciprofloxacin, Cefotaxime	1 (4.5)
**Environmental isolates**		
ER_1_	Ampicillin, Nalidixic acid, Cotrimoxazole, Erythromycin	1 (50)
ER_2_	Ampicillin, Nalidixic acid, Cotrimoxazole, Erythromycin, Chloramphenicol	1 (50)

Note: CR-Clinical resistant type, ER- Environmental resistant type.

Similarly two different resistant types were observed among *V. cholerae* isolates from environmental specimen. Fifty percent (n = 1) was of the environmental resistant type 1 (ER_1_) profile which was found to resist antibiotics such as ampicillin, nalidixic acid, cotrimoxazole and erythromycin. The remaining 50% showed an ER_2_ type (Table 3).

### Multidrug resistant *V. cholerae* and detection of *ctx* gene

**Figure 1:**
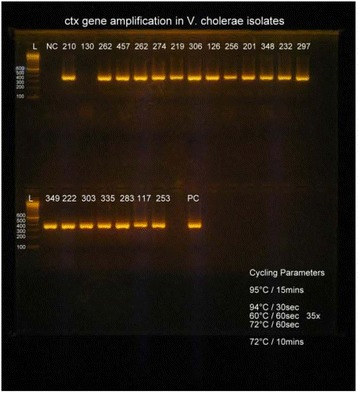
**Amplification of** ***ctx*** **gene in** ***V. cholerae*** **isolates (L-Ladder, PC-Positive control, NC-Negative control, clinical** ***V. cholerae*** **isolate; 210, 130, 262, 457 202, 274, 219, 306, 126, 256, 201, 248, 232, 297, 349, 222, 303, 335, 283, 117, 253 showing positive** ***ctx*** **band).**

**Figure 2:**
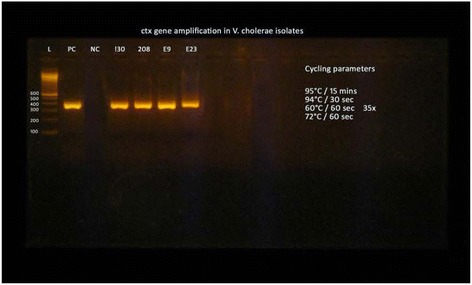
**Amplification of** ***ctx*** **gene in** ***V. cholerae*** **isolates (L-Ladder, PC-Positive control, NC-Negative control, clinical** ***V. cholerae*** **isolate; 130, 208 and environmental isolates; E9 E23 showing positive** ***ctx*** **band).**

All of the isolates were found to be multidrug resistant (Table 2) and highly pathogenic strains possessing the *ctx* gene of approximately 400 base pairs (Figures 1 and 2).

## Discussion

Cholera is one of the most predominant diarrhoeal diseases in Nepal even these days. In this study, we found evidence of *V. cholerae* in 4.9% of cholera cases among patients with diarrhoea and in 6.67% of drinking water samples. A study by Karki and Tiwari in Kathmandu reported 25.1% cholera cases in 2004 [20] and study by Tamang *et al.* in Kavre reported 31% of positive cases for *V. cholerae* in the same year [21]. The frequencies of *V. cholerae* among patients with diarrhoea were found to be still higher in the studies carried out by Kansakar *et al.* (11.17%), Karki *et al.* (27.1%) and Shah *et al.* (8.21%) [22-24]. The higher rate of the pathogens in the previous studies might be due to the hospital based analysis, however the prevalence may be lower among community based studies. The diarrhoeal cases were not only due to *V. cholerae* in our study. This study also reported 4% of diarrhoea caused by *Shigella* spp (*Shigella flexneri;* n = 14 *and Shigella sonnei;* n = 4) and 1.33% was due to intestinal parasites (*Entamoeba histolytica*; n = 3, *Cyclospora cayetanesis*; n = 2 and *Blasocystis hominis*; n = 1). The detail of results was not shown here. The predisposing factors such as poor sanitation, lack of safe drinking water and unhygienic foods preparations were found to be responsible for the repeated occurrence of the pathogens in Kathmandu and other districts of Nepal.

*Vibrio cholerae* El Tor O1 Ogawa was responsible for the endemics in Nepal before 2012 [21,24] and previous outbreaks of cholera in Kathmandu valley in 2004 [25]. In contrast, all of the isolates in this study were the *V. cholerae* O1 serogroups Ogawa and the classical biotype. Infections with classical strains are generally more severe than those with El Tor strains [6]. Three strains; *V. cholerae* O1 biotype El Tor, *V. cholerae* O1 biotype Classical and *V. cholerae* O139 have been frequently isolated in cholera outbreaks in Asian countries [26]. Although classical *V. cholerae* O1 caused the fifth and sixth pandemics, and presumably the earlier pandemics, the seventh pandemic was attributed to the El Tor biotype, which has been replaced by the classical biotype in this study. The Inaba and Hikojima sero subtypes were not found in this study. Other research conducted in Nepal had reported the occurrence of both Ogawa and Inaba serotypes with an interval of several years [21,27-29]. Children and the elderly people are mostly affected by cholera [2,30,31]. Contrary to this, adult populations of age group 20–30 years were highly infected accounting for 8.7% as compared to all other aged groups (3.4%) in our context (p = 0.018). The studies by Kansakar *et al.* [22] and Yadav *et al.* [32] found similar results in which most of the infected patients were adults aged 20 to 29 years and 15 to 29 years respectively. The greater incidence of infections in these groups was found because of their food habits outside the home including consumption of street food.

All the strains in this study were resistant to nalidixic acid, cotrimoxazole and ampicillin suggesting these drugs should not be used in the treatment of cholera. Das *et al.* also reported 100% resistance to the above three antibiotics [33]. A high incidence of cotrimoxazole resistant *V. cholerae* O1 strains has been reported in the studies in Africa, Asia and South America [34,35]. The study by Karki and Tiwari [20] found that all the *V. cholerae* strains were resistance to ampicillin while 97.8% isolates were susceptible to ciprofloxacin. Generally, fluoroquinolones have excellent activity against cholera [20] however; fluoroquinolone resistant strains of *V. cholerae* have recently been reported from India [33,36,37]. The majority of *V. cholerae* strains in our study were susceptible to tetracycline (100%), ciprofloxacin (90.9%), cefotaxime (81.8%) and chloramphenicol (90.9%) which may be effective alternative drugs for the treatment of cholera. However the development of resistance needs to be monitored. A similar result was also found by Shah *et al*. [24] showing sensitivity of 90% and 77.3% to cefotaxime and chloramphenicol respectively. However they showed that 81.8% of strains were resistant to tetracycline. Garg *et al.,* reported high-level resistance to chloramphenicol in India. This result contrasted to our findings [13]. Macrolide resistance was rarely reported in the studies by Harris *et al*., 2012 and Kanskar *et al*., 2011 [2,22], yet a high level erythromycin resistance (90.9%) was found in our study. Resistance to erythromycin and other antimicrobial agents among *V. cholerae* can be acquired through selected mutations over the time, or due to widespread use of antibiotics for prophylaxis in asymptomatic individuals [38].

All *V. cholerae* were found to be multidrug resistance in the study. MDR cholera epidemics have been reported from Bangladesh [39], Pakistan [40] and Nepal [9,23]. Indiscriminate use of antibiotics in the treatment of cholera and other enteric diseases has led to the emergence of antibiotic resistance among *V. cholerae*. Epidemics of MDR cholera (both classical and El Tor biotypes) have been reported worldwide [41]. MDR in *V. cholerae* can be attributed to either a spontaneous mutation or to the horizontal transfer of resistance genes between members of gut coliform or other co-existing microflora and *Vibrio* spp [42].

All *V. cholerae* strains tested in our study possessed the *ctx* gene. Toxigenic strains of *V. cholerae* contained the essential genetic element, CTX [43,44]. The isolates in the study were thus confirmed as toxigenic strains. The *ctx* genes are located in the CTX element and encode the cholera toxin CT. This toxin is primarily responsible for the severe secretory diarrhoea in infected person. Thus we screened all the isolates for the presence of *ctx* gene. Our results showed the presence of *ctx* gene of approximately 400 bp (~385 bp) in all the tested strains similar as described by Patrick *et al.* [19]. Similar genes were also detected in the environmental isolates. Chakraborty *et al.* [45] also found the critical virulence genes in the environmental strains of *V. cholerae*.

## Conclusions

*V. cholerae* is one of the major agents associated with diarrhoea outbreaks in Nepal with highest propensity during the rainy seasons. The burden of diarrhoea depends upon the strain and not all *V. cholerae* are toxigenic and epidemic. So, it is suggested that *V. cholerae* regularly be examined for the presence of the *ctx* gene from clinical and non-clinical samples to ensure identification of the toxigenic strain. Proper antibiotic susceptibility testing of *V. cholerae* is important to guide appropriate antimicrobial therapy.

## Abbreviations

AST: Antibiotic Susceptibility test
ATCC: American Type Culture Collection
CLSI: Clinical Laboratory Standard Institutes
CR: Resistant profile of Clinical *V. cholerae* isolates
CT: Cholera Toxin
*Ctx*: Cholera toxin producing gene
DNA: Deoxyribonucleic Acid
ER: Resistant profile of Environmental *V. cholerae* isolates
KCMS: Kantipur College of Medical Sciences
KTM: Kathmandu
LB: Luria Bertani
MDR: Multi Drug Resistance
NHRC: Nepal Health Research Council
RLABB: Research Laboratory for Biotechnology and Biochemistry
TCBS: Thiosulphate Citrate Bile Sucrose Agar
UGC: University Grants Commission
PCR: Polymerase Chain Reaction
